# Deep learning techniques for detection and prediction of pandemic diseases: a systematic literature review

**DOI:** 10.1007/s11042-023-15805-z

**Published:** 2023-05-29

**Authors:** Sunday Adeola Ajagbe, Matthew O. Adigun

**Affiliations:** 1 Department of Computer & Industrial Production Engineering, First Technical University Ibadan, Ibadan, 200255 Nigeria; 2 Department of Computer Science, University of Zululand, Kwadlangezwa, 3886 South Africa

**Keywords:** Artificial intelligence (AI), Deep learning (DL), Infectious diseases (IDs), Machine learning (ML), Optimization techniques, Pandemic

## Abstract

Deep learning (DL) is becoming a fast-growing field in the medical domain and it helps in the timely detection of any infectious disease (IDs) and is essential to the management of diseases and the prediction of future occurrences. Many scientists and scholars have implemented DL techniques for the detection and prediction of pandemics, IDs and other healthcare-related purposes, these outcomes are with various limitations and research gaps. For the purpose of achieving an accurate, efficient and less complicated DL-based system for the detection and prediction of pandemics, therefore, this study carried out a systematic literature review (SLR) on the detection and prediction of pandemics using DL techniques. The survey is anchored by four objectives and a state-of-the-art review of forty-five papers out of seven hundred and ninety papers retrieved from different scholarly databases was carried out in this study to analyze and evaluate the trend of DL techniques application areas in the detection and prediction of pandemics. This study used various tables and graphs to analyze the extracted related articles from various online scholarly repositories and the analysis showed that DL techniques have a good tool in pandemic detection and prediction. Scopus and Web of Science repositories are given attention in this current because they contain suitable scientific findings in the subject area. Finally, the state-of-the-art review presents forty-four (44) studies of various DL technique performances. The challenges identified from the literature include the low performance of the model due to computational complexities, improper labeling and the absence of a high-quality dataset among others. This survey suggests possible solutions such as the development of improved DL-based techniques or the reduction of the output layer of DL-based architecture for the detection and prediction of pandemic-prone diseases as future considerations.

## Introduction

The global economy has collapsed due to the coronavirus (COVID-19) pandemic [1, 22, 41]. New strain variants, a lack of social self-control, and optional vaccination all increase the likelihood that COVID-19 will persist and behave like a seasonal sickness. All nations are developing plans to gradually resume their economic and social activities since the socioeconomic situation has grown unsustainably [6, 79]. The COVID-19 pandemic prompted countries to impose strong limitations throughout 2020, portraying a scenario of decreased hospital visits that is unprecedented and the use of artificial intelligence (AI) and the internet of things (IoT)-based on the management of the pandemic. AI-based approaches to analyze, detect, classify and predict the trend of the deadly disease were developed [42].

**Fig. 1:**
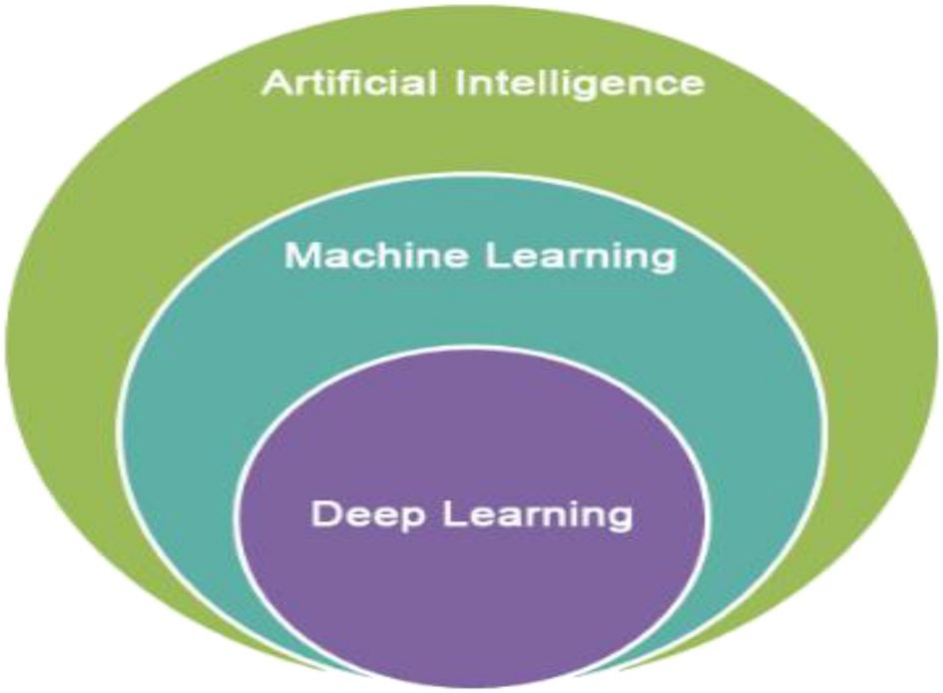
AI/ML/DL Relationship

Since its inception in 1956, AI has been studied to create “intelligent agents”—devices that can sense their surroundings and respond in ways that increase the possibility that they will succeed in attaining their objectives [35]. AI in healthcare started with the creation of expert systems, which were based on rules gleaned from expert interviews, and then translated and programmed [71]. This expert system involves the use of AI for the analysis, learning and deduction of inference from data. AI techniques have three methods, they are DL, ML and AI itself. The relationship between AI, ML, and DL is depicted in Fig. 1 [3].

The terms AI, ML, and DL are all somewhat confusing nowadays, DL is a subset of ML, which is a subset of AI, which is a catch-all phrase for any intelligent computer program. In other words, all ML is AI, but it is not all AI is ML and ditto DL. In the domains of computer science, data analysis, software engineering, and AI, ML and DL represent a significant evolution [10]. The study of AI is an active topic of study with many promising areas for further research including the detection and prediction using various techniques and applications [86]. Manual detection of pandemics and infectious diseases (IDs) is quite difficult because it takes a lot of time and is prone to human error. The potential of AI techniques is being further explored in the creation of automated and precise COVID-19 detection and prediction systems. Convolutional neural networks (CNN), in particular, have grown significantly in prominence among AI approaches for the detection, classification, diagnosis, prediction and other healthcare-related issues [27, 57, 84].

The continuing new Coronavirus (COVID-19) epidemic has caused millions of infections and many fatalities globally [56]. The World Health Organization (WHO) declared COVID-19 a global pandemic on 2020, the WHO is the only recognized body to declare IDs or any diseases to be a pandemic [45]. In the past, a lot of scholars have tried to forecast a COVID outbreak and its consequences [45]. Some people believe that time-series variables are the primary factors that can affect the onset of viral infections such as influenza and severe acute respiratory syndrome (SARS) [13, 67]. Worldwide, numerous IDs pose a threat to life. While certain viruses, like MERS, generate pandemics by moving from one country to another and across continents, others, like Ebola, circulate only in a small number of nations. Numerous studies have been done to quantify and forecast the occurrence of IDs as a result of the global epidemic of IDs [55].

IDs are infections that have suddenly developed in a community or that have existed but are now spreading quickly over the globe. IDs include the following: known infections spreading to new populations and areas; the revelation that an infection is the primary cause of a known disease; A new infection brought on by changes in the microorganisms, previously undiscovered infections emerging in locations whose habitat is changing; and an old infection resurfacing because it is no longer treatable or as a result of a failure in public health systems. When IDs transient a geographical region (country), it becomes a pandemic [61].

Rohr et al. (2019) [80] presented the links between Outbreaks of illnesses in humans and massive food distribution Infectious illnesses are spreading at an astonishing rate, wreaking havoc on global economics and public health. Thus, the social and environmental variables that give birth to microbial pathogens, as well as management techniques that may lower the likelihood of disease emergence or re-emerging, are of special importance [53]. At the same time, undernutrition which is a lack of one or more nutrients, continues to be a major cause of worldwide disease. Infectious illnesses are spreading at an astounding level over the world, while global food consumption is expected to skyrocket by 2100. The paper summarizes the methods through which anticipated agricultural growth and intensification will affect human infectious illnesses, as well as how human IDs may affect food production and distribution.

The study employs the systematic literature review (SLR) approach to study the trend of research in DL techniques for the detection and prediction of pandemics, SLR was chosen because it is based on well-defined methods and aims to find, assess, and aggregates all relevant evidence/ literature about the subject matter in a fair, repeatable and auditable manner. The following are the objectives of this survey:

i. Study the trend of the pandemic since its inception globally;
ii. Identify the technological innovations available through the use of AI techniques in the detection and prediction of pandemics;
iii. Study the significance of DL techniques for detecting and predicting pandemics and;
iv. Identify the limitations of the existing DL techniques in the detection and prediction of pandemics
v. To ameliorate the open issues with the key strength of DL techniques for potential scholars to benefit from the open issues.

This research is organized as follows. Section 2 presents the technologies for the detection and prediction of pandemics, this includes data capturing technologies, pandemic data analysis and pandemic management technologies. Section 3 presents the survey methodology, while Section 4 contains the results and discussion of the survey, and Section 5 concludes the survey and presents future considerations to realize an efficient DL technique for the detection and prediction of pandemics.

## Technologies for the detection and prediction of pandemic

Technologies play an important role in pandemics and IDs detection and prediction. The IoT can help by creating an early warning system to stop the spread of dangerous diseases. Integrated IoT networks, advancements in data analytics, AI, and universal networking on a worldwide scale are yet required to achieve this. These have been extremely beneficial for many aspects of humanity, especially in terms of preventing contagious diseases. A global network of IoT sensors in the healthcare sector offers both short-term and long-term benefits. Healthcare professionals and legislators have been able to follow any person who has been “compromised” as they pass through border controls. This would make it possible to focus on quarantine and, provide quick care, which would stop the coronavirus and other dangerous diseases from spreading. Long-term negotiations for the creation of a global early warning system should begin between large multinational organizations like the WHO and the United Nations. Such a tool could identify IDs before they spread globally. Worldwide emergencies, like the coronavirus, cause a number of fatalities, heightened stock price volatility and instability. A global detection system will clear up this uncertainty and give decision-makers the financial chance to react rapidly to pandemics and emergencies affecting all aspects of public healthcare [7].

### Pandemic management technologies

Digital technology’s involvement in pandemic containment can take numerous forms and deliver great benefits. Thus, big data and AI research are linked to the pandemic that has plagued the world for decades (for instance the Spanish flu, SARS, MERS, avian flu, Ebola, and SARS-COV-2) as a fascinating and significant study stream. Along with the widespread use of digital health technology, another intriguing phenomenon relating to the collective activities done by the many players participating in the Healthcare Ecosystem may be seen in the management of the technologies for data collection, diagnosis, spread control and many more [50, 81]. Specialists, virologists, supranational agencies, ministries, hospitals, device suppliers, and all other entities working to minimize pandemic transmission are organizing their operations. While the necessary measures include striving to cooperate with quarantine, epidemic clusters, and country lockdowns owing to the lack of a viable particular vaccine. Blockchain technology, IoT, robotics and other related technology assisted have been helpful in the management of the ongoing pandemic [11].

The massive amount of complicated data from various sources about the number of patients, IDs contacts, mortalities, and so on has necessitated the use of big data technology to facilitate timely recognition of patient profiles, recurring and comparable trends of evolving disease actions, use and reaction to therapies, and so on. The use of big data in the administration of the pandemic, as well as the application of prediction algorithms, will be crucial in understanding the pace of viral transmission and the population at risk, as well as the natural history of the infection, high fatality rate, as well as the most effective treatment choices and preventative and control strategies [85].

Nguyen et al. (2020) [72] proposed a framework for COVID-19 detection based on data from smartphone sensors like webcams, microphones, heat, and sensor systems. The ML algorithm was employed in Italy, as well as many other nations throughout the world, to learn and gather information about illness symptoms based on collected data. In comparison to standard medical kits or skilled scanners, this strategy provides a low-cost and quick way for coronavirus screening since data inferred from sensing devices may be used effectively in a variety of specific applications.

AI technologies show significant effectiveness in assisting decision-makers in the virus management process. Allam and Jones (2020) [15] urged the use of AI and data-sharing regulatory mechanisms, to improve worldwide knowledge and control of urban health during the COVID-19 epidemic. For instance, when AI is combined with IoT devices deployed in many smart cities for early epidemic detection, further benefits can be realized. When medical data is gathered and distributed throughout and within smart cities. Rao and Vazquez (2020) [77] proposed a phone-based online questionnaire to collect people’s travel histories and common indicators. The acquired data may be evaluated using ML algorithms to study and predict the risk of infection, allowing for the early identification of high-risk cases for isolation. It limits the virus’s propagation to susceptible persons. In a recent review of DL detection research applications, challenges, and future directions by [68]. The study focused on the review of DL applications in healthcare featuring abdomen, cardiac, pathology, retina and diabetic retinopathy. The review did not consider the detection and prediction of pandemics.

### Pandemic data capturing technologies

An innovative community-wide monitoring method called wastewater-based epidemiology (WBE) provides thorough real-time data on the state of public and environmental health and can support public health initiatives, such as those aimed at preventing IDs outbreaks. A potentially affordable method of keeping track of disease progression through WBE to stop local outbreaks is the use of Biosensors. In order to avert future pandemics, [52] reviewed the technical and economic viability of 18 recently developed Biosensors for the detection of IDs pathogens in wastewater. Municipalities lacking centralized laboratories, however, are probably still unable to analyze WBE samples.

Due to the paucity of reliable knowledge regarding the disease and its behaviors, detecting and predicting pandemic and IDs is a particularly difficult undertaking. Recent research has looked into several ways to prevent pandemics and infections effectively using IoT sensors. For the detection and prediction of pandemics and IDs, numerous researchers use the IoT to gather real-time sensory input data. One of [65] main investigations was a set of sensors that were dispersed across the workplace to look for or acquire information about pandemics and IDs. Real-time data could be captured by sensors and stored in the cloud, but the user would need to be made aware of the actual circumstances surrounding the data. The obtained data is subjected to filtering and analytics algorithms, which extract the data in the form of user information. Meraj, et al., (2021) [65] discussed COVID-19 as the most recent pandemic while also discussing the flu, zika, and H1N1. The research inquiry of the document also focused on the method of Remote Excess of Experts with IoT data.

### Pandemic data analysis technologies

Various technologies were used to analyze pandemic data such as data mining, AI, ML, DL, IoT, and Blockchain among others. Kumar, Misra, & Chan (2022) [58] presented diagnostic technologies to analyze the coronavirus disease pandemic. In contrast to earlier coronavirus outbreaks of SARS and Middle East Respiratory Syndrome, chest X-ray has not shown adequate sensitivity to be useful in first-line screening techniques. Despite the fact that current national and international guidelines propose using *reverse transcription polymerase chain reaction (*RT-PCR) as the initial screening technology for identified COVID-19 infections, academic and regional procedures must reflect local resource allocation when releasing general guidelines. So far, successful containment and social mitigation techniques have relied on united governmental reactions, even though the underlying philosophies of these activities may not be universally applicable in many Western nations. As the need for radiology personnel grows, preliminary findings point to a prospective role for machine-learning techniques as risk prediction criteria.

Pan et al., (2020) [75] opined during the recovery from the coronavirus pulmonary outbreak, studying the temporal course of lung alterations on chest CT was studied. Patients with RT-PCR proven COVID-19 infection were included in the retrospective study. Patients who experienced significant respiratory distress and/or required oxygen at any point throughout the disease’s progression were excluded. Repeat chest CTs were acquired at 4-day intervals. The overall CT score was calculated as the sum of the lung activity indicated. Twenty-one individuals with COVID-19 pneumonia were assessed. These patients had a total of 82 lung CT scans and were all released after an average of 17 days in the hospital. The degree of lung irregularities on chest CT in the individuals recovering from COVID-19 pneumonia was greatest roughly 10 days following the beginning of symptoms.

Chakraborty et al. (2020) [32] presented a DL classifier sentiment analysis of COVID-19 pandemic tweets. People are fearful of panic, terror, and worry as the number of instances increases exponentially over the world. The physical and mental health of the global populace has been shown to be inversely correlated with this pandemic illness. The study stressed the fact that tweets comprising all handles associated to COVID-19 and WHO have been ineffective inappropriately advising people around this pandemic epidemic. The study indicated that, despite the fact that individuals tweeted primarily nice things about COVID-19, netizens were preoccupied with retweeting the bad tweets, and that no beneficial terms could be located in WordCloud or calculations based on word frequency in twitter posts. The claims were confirmed using a suggested model that included deep learning models with allowable accuracy of up to 81%. Aside from this, the researchers have suggested the use of a fuzzy rule based on a Gaussian membership function to accurately identify attitudes in tweets. The accuracy of the aforementioned model is up to an acceptable rate of 79%.

Oh, et al. (2022) [73] applied in low COVID-19 occurrence circumstances, neighborhood-scale wastewater-based epidemiology was used. WBE is a new technique for neighborhood COVID-19 monitoring, it has been studied largely at major sewer sheds such as wastewater treatment facilities that serve a massive community. From January to November 2021, WBE was applied to seven neighborhood-scale sewer sheds. Data from society testing revealed a mean occurrence of 0.004% in these sewersheds (97.0% of monitoring periods indicated two or less daily illnesses). The research findings indicated that neighborhood-scale WBE can detect local epidemics that city-scale WBE would miss. As a result, the author’s findings indicated that neighborhood-scale WBE was an effective community-wide disease surveillance technique while pandemic incidence remains low.

Zheng et al. (2022) [91] presented influencing variables and coronavirus clustered features. The study used descriptive statistics and regression analysis on COVID-19 data from several nations to analyze and assess various regression models. The extreme random forest regression model performed best, and parameters like population size, ozone, average age, life span, and human development index were found to have a moderate influence on the dissemination and dispersion of COVID-19 in the ERFR-based method. The spectral clustering technique was used to examine the pandemic clustering properties. The spectrum clustering visualization findings revealed that the geographical distribution of worldwide pandemic spreading creation was highly clustered, and its grouping features and impacting variables also demonstrated some uniformity in dispersion.

Oyelade, & Ezugwu (2020) [74] presented a case-based reasoning approach for new coronavirus identification and diagnosis. The study applies an upgraded CBR model for cutting-edge reasoning tasks in the categorization of suspected COVID-19 instances. For case similarity calculation, the CBR model makes use of a unique selecting features and the semantic-based computational formalism introduced in this paper. The database was first populated with 71 cases received from the Italian society of medical and interventional radiology (SIRM) repository. The results showed that the suggested technique in this study correctly sorted reported cases into respective categories with a 94.54% accuracy. The study discovered that the suggested approach can assist clinicians in readily diagnosing probable cases of COVID-19 based on patient medical records without the samples being sent to laboratory testing.

### Deep learning for detection and prediction using COVID-19 dataset

The manual detection of the infection using radiographic pictures is quite difficult since it takes a lot of time and is very prone to human error [28]. The development of automated, precise, and effective methods for pandemics and Identities, including COVID-19 detection, has showed promise and is now being pursued. For the categorization of COVID-19, DL-based strategies have been increasingly prominent among AI technologies [6]. The proposed system included three stages: preprocessing with lung segmentation, removing the environment that did not provide relevant information for the task and could lead to biased results; classification model trained under transfer learning scheme; and finally results analysis and interpretation via heat maps visualization. The most accurate models attained a 97% COVID-19 detection accuracy [23].

A recent study suggested an automatic CNN-based DL-based classification technique that showed a quick COVID-19 detection rate. 3616 COVID-19 chest X-ray pictures and 10,192 images of healthy chests that were later enhanced make up the training dataset. The COVID-19 symptoms were initially identified utilizing the dataset by applying eleven pre-existing CNN models. The potential of MobileNetV2 made it a candidate for further improvement. Of all the deployed CNN models, the final model produced the greatest accuracy of 98% in classifying COVID-19 and healthy chest X-rays. The findings imply that the suggested strategy outperforms existing ones in accurately detecting illness symptoms from chest X-ray pictures [12]. A DL-based method to categorize COVID-19 based on x-ray images was recently introduced in a study. The results were encouraging and might be used to distinguish infected individuals from healthy individuals. The Kaggle dataset of COVID-19 X-ray pictures was used for the trials, which were carried out using a ResNet50 DL-based approach with 5 and 10 folds cross-validation. The experiment’s findings demonstrated that 5 folds had an accuracy rate of 97.28%, which was higher than 10 folds [4].

A recent study examined and contrasted various DL-based enhanced approaches used to detect COVID-19 in X-ray and CT-scan medical pictures. VGG16, DenseNet121, ResNet50, and ResNet152 were four pre-trained CNN models that were employed for the binary classification job for the COVID-19 CT-scan. The appropriate architecture, pre-processing, and training settings for the models will be determined largely automatically via the Fast.AI ResNet framework. The F1-score and accuracy for the diagnosis of COVID-19 using CT-scan images were both over 96%. In order to overcome the lack of data and shorten the training period, we also used TL-based techniques. X-ray image tasks were classified in binary and many classes using the modified VGG16 deep transfer learning architecture [90].

## Methodology

**Fig. 2:**
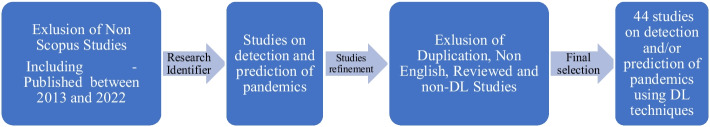
The chart for analyzing research findings and the selection criteria

This section describes the process used to locate articles and how those articles were used to determine the specified objectives in relation to the selection criteria and the solutions to the research questions. The four goals of the current survey were established through four separate research questions (RQ) [17]. Figure 2 shows the selection chart and criteria for research surveys. This section proffers the review guide that anchored SLR principles in this survey.

- RQ1: What has been the pandemic experience globally?
- RQ2: How far has AI/DL/ML techniques contributed to the detection and prediction of pandemics?
- RQ3: How has DL techniques contributed to the detecting and predicting pandemics?
- RQ4: What are the limitations of the existing DL techniques in the detection and prediction of pandemics?

### Search techniques

The references for this review were discovered through Scopus searches. This comprises journal articles, chapters from conferences that were referred, and other types of content from top research databases that Scopus indexed between 2013 and 2022. Because they were regarded as trustworthy databases for study in AI, computer science, and biomedical sciences in general, these databases were chosen. These include Sensors, IEEE Access, Computers in Life and Medicine, Electronics Switzerland, Computer Materials a Continua, and Applied Sciences. We incorporated items from January 2013 through September 2022 that were found using these searches.

### Study selection/ studying choice

Both prospective and retrospective studies that reported on original English-language research on the detection and prognosis of pandemics were taken into consideration. Reports on the development and application of DL approaches for pandemic detection and prediction are available. To concentrate on the research work with in-depth knowledge of the study area and studies with the detection and prediction of pandemics using information not currently available in routine clinical care or with results unrelated to the typical care provided by physicians, conference papers, journal articles, and chapter contributions were included, with the exception of one. Others were excluded based on our selection criteria.

### Data analysis in the selected articles

Articles were first chosen based on sections 4.1 and 4.2. Three concepts (detection, prediction, and pandemics) appeared in the titles, abstracts, and keywords of the publications we obtained. Each abstract, type of pandemic/dataset, methodology, and performance evaluation used, as well as the outcomes obtained, were researched and evaluated. Publications using DL techniques were carefully examined and analyzed. 44 papers from the final portfolio were subjected to a bibliometric and content analysis, which grouped the research’s findings and observations.

#### Inclusion criteria

Published studies in English no sooner than January 2013 and no earlier than October 2022. Research Gate, Semantic Scholar, PubMed, Google Scholar, and Science Direct published databases.

#### Exclusion criteria

Types of published papers (review, research, letter and journals). The search did not include the reviewed or non-DL papers.

Duplication: Duplicated papers were excluded from the search results.

Time of publication: The time frame for the published research was ten years, from 2013 to 2022.

Language of publication: English was employed as the study’s official language and as a qualification for inclusion.

Process for the Research: From 2013 to 2022, the research was conducted by hand-searching certain journal articles. The following research methodology was applied to this review:

i. Resources: The process for the research includes journal articles published between January 2013 and October 2022. In addition, papers that used DL for the detection/prediction of pandemics in the last ten years (2013-30th of September 2022) among these were further investigated. Thus, references of all selected journal articles were checked to see if pertinent papers had been selected.
ii. Research Keywords: The following keywords were utilized in the research questions for this review: Detection, prediction and pandemic.
iii. Research Expressions: For this review, the guidelines for utilizing the research words have been followed: Keywords terms are taken from research questions related to the pandemic using DL techniques. The AND logical operator was used to group keywords into categories, and the OR logical operator was used to combine phrases and synonyms.

## Results and analysis

### General outcome of the literature search

**Fig. 3:**
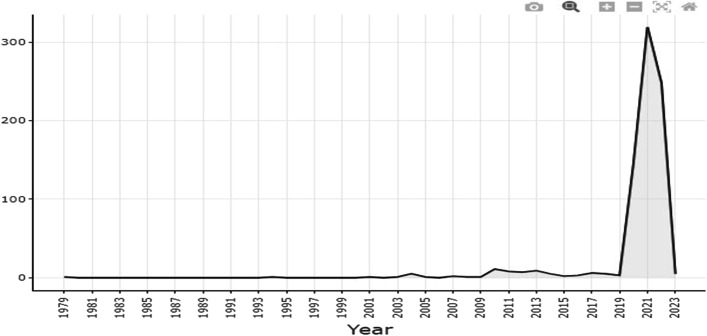
Trend of the detection and prediction of pandemic publications

**Fig. 4:**
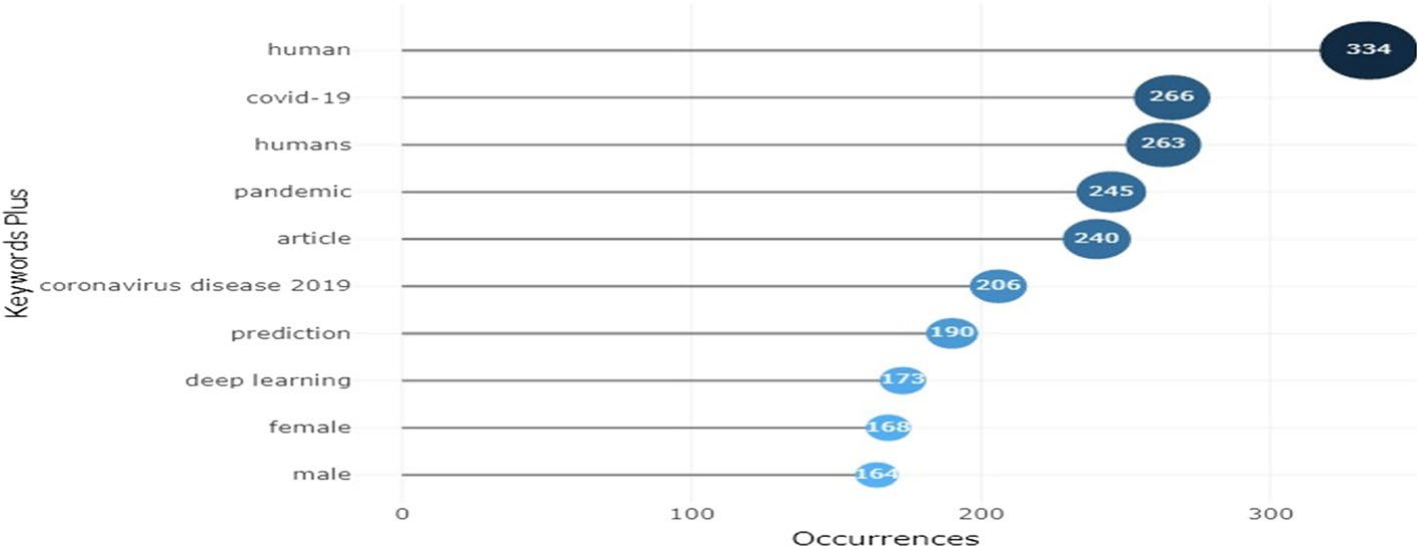
Most frequent words

A total of 790 research papers were extracted using the stated search terms. The statistics of these papers are presented in Table 1, which covers the journal article, conference papers, chapters in books, letters editorials and so on. Each of the paper titles was examined independently using the inclusion and exclusion criteria, leaving 44 papers that either work on pandemic detection or prediction or both. 746 publications were deleted because they had no relevance to the research subject (some were excluded because they were focused on other aspects of detection and prediction non-specific to the pandemic, some are pandemic studies but they are bot detection and/or prediction). Before these 8320 were detected, they were further reviewed based on their abstracts, introductions, and conclusions and found none relevant to the subject area. 4 duplicate papers were removed using Endnote X8. Several articles were eliminated because they were difficult to understand in their entirety or because their abstracts showed that they had no bearing on the question. A total of 44 papers were selected for journal article assessment and state-of-the-art review; each was examined in its completeness, separately, and again using the inclusion and exclusion criteria. Figure 3 shows the graphical representation of the number of publications received and published before and after the COVID-19 pandemic. The graph shows that the highest publications on the pandemic were published in the year 2021. Figure 4 presents the most frequent word led by humans and followed by COVID-19, DL also appears making it a prominent AI technique that is contributing to the detection and prediction of pandemics.

**Table 1:** Classification of Publication by document type

Document Type	Number
Article	442
Conference Paper	159
Conference Review	73
Review	68
Book Chapter	27
Note	8
Letter	6
Editorial	4
Book	2
Erratum	1
Total	790

### Outcome of our findings based on research questions

Some of the selected articles uniquely answered the questions by addressing one unique facet of Pandemic detection and prediction based on search area, application area, and geographical area among others. While other articles mostly re-emphasized what was written in other articles.

#### The global trend of the pandemic

##### RQ1: What has been the pandemic experience globally

**Fig. 5:**
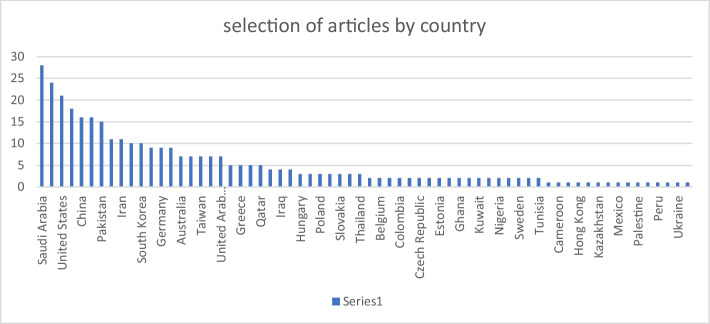
Trends in the number of publications using author country as a search key between January 2013 to September 2022

**Fig. 6:**
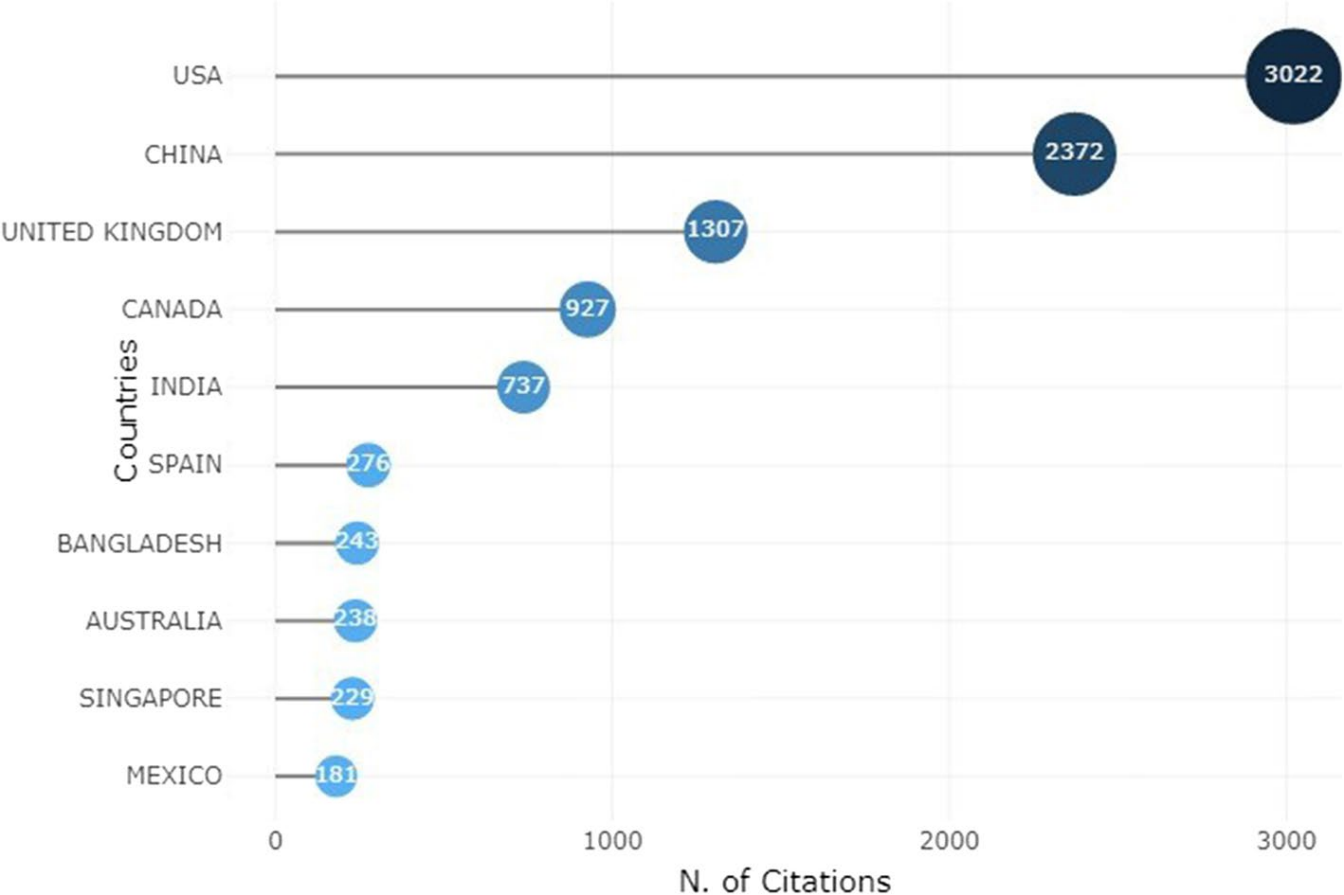
Ten most cited countries

Presenting the statistics from the evaluated literature, the global trend of pandemic based on the review literature and using selected search terms in Scopus in October 2022 is the focus of Fig. 5 showing the trends of the pandemic, Saudi Arabia, United States, China, Pakistan and Iran are top five (5) countries with cases of pandemic globally. Conversely, Kasakhstan, Mexico, Palestine, Peru, and Ukraine are at least five (5) countries with cases of pandemic globally. It is worthy of note that the studies on DL techniques and prediction increased geometrically between 2019 and 2022, this may not be unconnected to outbreak of COVID-19 pandemic. Meanwhile, many countries in Africa that has no sophisticated healthcare infrastructure needs to prepare for eventualities, many developed countries were able curtailed the pandemic because of their sophisticated healthcare facilities. These answered (RQ1). To further explore the pandemic experience globally, the most cited countries in the reviewed publications, was investigated and the outcome is presented in Fig. 6 as the most cited countries. The United State of America is leading followed by China.

In answering RQ1, the pandemic, especially the COVID-19 pandemic continues to impact all aspect of lifes which is affecting 288 countries and territories globally. COVID-19 as one the pandemic that has affected the world infected over 626,360, 380 people globally while 6,560,192 deaths have been recorded as of 8th October 2022 [89]. The pandemic has disproportionately hurt the weak and the poor, and it poses a threat to further impoverish millions of people. The pandemic caused a lockdown and shutdown of global business which resulted in the current high inflation rate in the global market. In terms of publiccation, major attention was shifted to the COVID-19 pandemic which cause the rapid increase in COVID-19 articles in the last three (3) years, although the present study covers ten years of the survey.

#### Technological innovations available for the detection and prediction of pandemics

##### RQ2: How far has AI/DL/ML techniques contributed to the detection and prediction of pandemics?

**Fig. 7:**
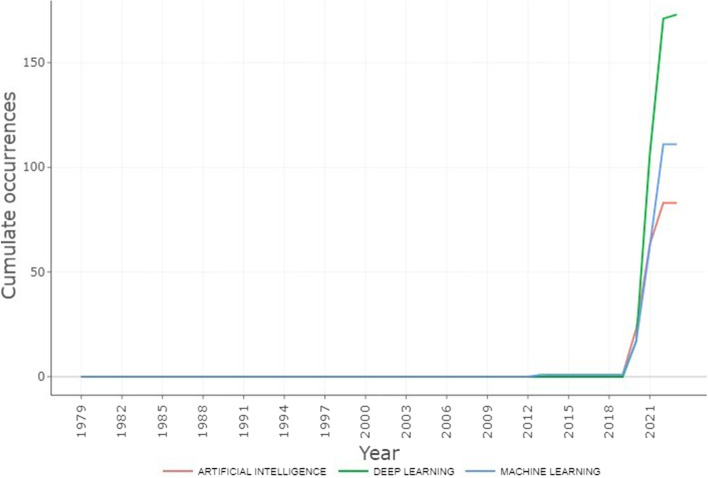
Comparison of the trend of using the three AI-based techniques used in pandemic

**Fig. 8:**
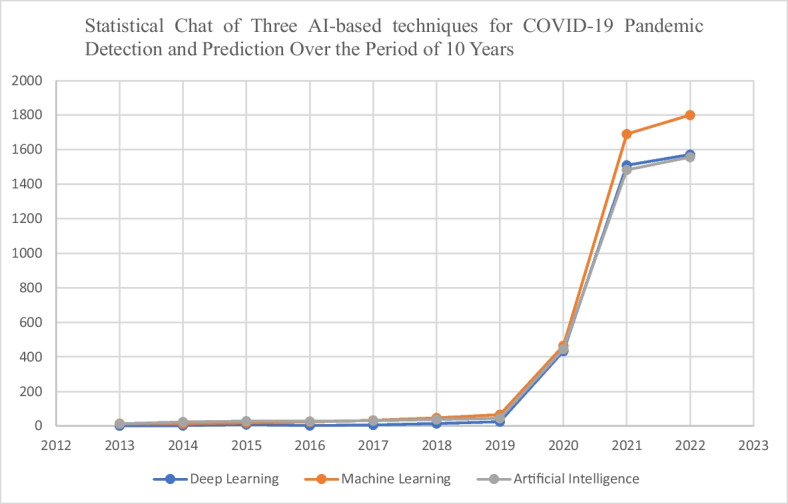
Statistical Chat of Three AI-based techniques for COVID-19 Pandemic Detection and Prediction Over the Period of 10 Years

Presenting the statistics of the literature reviewed, the trends in the number of publications using selected search terms in Scopus in October 2022 were the focus, Fig. 7 shows the comparison of the trend of using the three AI-based technologies publication in pandemic using selected search terms in Scopus for the period under review. These answered research question 2 (RQ2). In the analysis of technological algorithm in detection and prediction of pandemic over the period of ten (10) years. It comprises of AI/ML/DL techniques, DL techniques were the least AI-based technological used for pandemic analysis and studies, it was reported in 3573 publications, most of these publications were not focusing on the detection and prediction of pandemics. Figure 7 gives the details of the contributions of AI-based studies extracted from the database, it presents the comparison of three AI-based technological tools used in the analysis of pandemic data the last 10 years. Figure 8 presents the statistical chat of three AI-based technologies for COVID-19 pandemic detection and prediction over the period of 10 years in the current study.

#### Significance of DL techniques detecting and predicting pandemics

Although, DL was the least AI-based used among the three. This calls for further investigations. Hence, the need for RQ3, that inquires for the significance of DL techniques for detecting and predicting pandemics. DL is significant in the detection and prediction of pandemics, it was mentioned in 1840 publications and 11 fields or subject areas.

##### RQ3: How has DL techniques contributed to the detecting and predicting pandemics?

The analysis of DL-based state-of-the-art studies reviewed shows the novelty DL-based techniques in the pandemic detection and prediction. DL techniques were used for various purposes in the studies including the detection and prediction of pandemics. Table 2 presents the outcome of the state-of-the-art reviewed literature on DL for the detection and predictions.

**Table 2:** State-of-the-art Review of DL Studies Focusing on Detection and Prediction

Ref	Study goal(s)	Dataset	Techniques used	Optimization / Feature selection used	Results obtained	Inferences
[70]	Detection of fake news in Arabic language	a large and diverse Arabic COVID-19 fake news dataset locally constructed	DL and transformer models (ArBert, MARBert, Araelectra and QaribBert—Base)	None	Accuracy 98%, Precision 99.4%, Recall 98.83% and F1-Score 98.9%	The dataset was not primarily a pandemic dataset rather an NLP dataset
[5]	The study proposes PCovNet, a LSTM-VAE-based anomaly detection framework, for detection of COVID-19 infection	Covid-19 wearable device dataset	PCovNet, a LSTM- Variational Autoencoder (VAE)-based	NA	With average Precision, Recall, and F-beta scores of 0.946, 0.234, and 0.918, respectively, the framework’s result configuration identified RHR anomaly.	The results demonstrate the viability of deploying wearable technology equipped with such a deep learning framework as a secondary diagnostic tool to get around the COVID-19 presymptomatic identification issue. The outcome of this study might be improved if an optimization technique was introduced
[18]	The primary goal of this article was to detect the emotional impact of teaching strategies on students’ learning.	AffectNet facial recognition dataset emotion-related tags in English, German, Spanish, Portuguese, Arabic, and Farsi were used	Deep neural network (DNN) (ResNet59).	NA	Precision 0.95, F1-score 0.96 and recall 0.94	DL technique was used in the study for the detection of students’ emotions but the study did not in any way related to the pandemic study.
[43]	Detection of facial mask using DL	The dataset used consist 1915 images of people wearing masks and 1918 images of people without masks.	MobileNetV2	Adam optimizer was used	99.48% accuracy was achieved	Though this was an impressive outcome, but there is a need to confirm this outcome using other state-of-the-art metrics as accuracy alone cannot confirm the effectiveness of the method in this scenario
[59]	COVID-19 detection from lung CT-Scans was suggested	Covid-19 chest CT-scan images	A fuzzy integral-based CNN ensemble was proposed alongside DL Model: VGG-11, GoogLeNet, SqueezeNet v1.1 and Widely ResNet-50-2	NA	Accuracy and sensitivity of 98.93% each were achieved when evaluated	Other state-of-the-art metrics were not used to evaluate the model to ascertain its efficiency. Also, the model outcome could be improved through the use of feature selection and/or optimization techniques on the same dataset and proves to be a reliable pandemic detection technique(s).
[38]	COVID-19 IDs dynamics simulation model was proposed	COVID-19 dataset from IEEE and Kaggle databases	All-People-Test-Based method	NA	The outcome shown accuracy, precision, recall, F1-score of 99.02%, 99.0%, 99.0%, 99.0%	According to the authors, “there need for improvement, given the COVID-19’s importance, the proposed model has misclassified 0.98% of test data, which is not ideal.
[82]	COVID-19 detection using a novel ensemble classifier based on TL	Chest CT-scans dataset	Ensemble CNN-based model	NA	Accuracy, Precision, recall, F1-score and AUC measures of 98.99, 98.98, 99.00, 98.99, and 99.00 respectively	The outcome of this study might be improved if an optimization technique was introduced
[87]	The goal of the study was to estimate the percentage of COVID-19 infection rate.	The CT scan slices of patients either infected with COVID-19 or healthy provided by ICIAP 2021.	Deep Regression model	NA	The study showed a performance of m = 21 neighbors, with MAE = 1.847, MdAE = 1.119, PC = 0.994, RMSE = 2.838.	The study is unable to further investigate as framework in these findings is absent in the known DL-based framework. More Deep Learning methods should be used to get better accuracy in predicting future pandemics.
[49]	To increase the accuracy of CNN in classifying or detecting pathological voices	Voice datasets from Far Eastern Memorial Hospital was used	SincNet system was developed by replacing the first layer of CNN with Sinc filters and a front-end signal processor	NA	Accuracy 77.5%, Sensitivity 80.0%, Specificity 65.0%, UAR 72.5%	A voice dataset was used in this study but not a pandemic dataset
[9]	This research presented a DL-based approach to detection masks covering faces in public spaces.	Face mask dataset from Masked Faces (MAFA)	ResNet50V2	NA	Precision 0.97, Recall 0.97, F1-score 0.97, Inference Time (ms) 7, Training Accuracy 91.93 and Validation Accuracy 90.49%	Four DL-based techniques; ResNet50V2, VGG19, MobileNetV2 and InceptionV3 were used in the study for face mask detection. ResNet50V2 results appear good but better results can be achieved
[51]	The DL-based method for COVID-19 pandemic detection was proposed and Transfer Learning (TL) concept was incorporated	Chest X-ray images dataset was used	ResNet-based model	NA	ResNet model achieved an accuracy of 97%	The outcome of ResNet appear good but better results may be achieved when optimizer is used
[47]	A novel method to analyze, predict, and detect the COVID-19 pandemic was proposed.	Publicly available COVID-19 Chest X-ray dataset from Johns Hopkins University was used	CNN-model	NA	The outcome of the study was accuracy of, 96.51%, precision 97.67%, recall 97.73% and F1-score 97.20	For quick and easy forecasts of the COVID-19 infection, the study used the normal distribution. It also used least squares parameter curve fitting to estimate the parameters of Gaussian curves for multiple nations on various continents. Meanwhile, the outcome of the study might be improved if the authors have utilised optimization techniques of feature selection techniques’
[60]	The goal of the study was to categorize COVID-19 artefacts in altered real-world scenarios using chest X-ray images.	10,848 images make up a substantial and balanced dataset.	CNN technique was proposed for the classification of chest X-ray images.	A novel Bayesian optimization-based	Accuracy, 96.51%, sensitivity specificity	The study did classification using COVID-19 pandemic dataset, prediction was not considered in this study and utilization of an optimization algorithm might improve the results obtained.
[2]	The paper proposed a multi-level diagnostic framework for the accurate detection of COVID-19 using X-ray scans based on transfer learning.	The study was benchmarked on a COVID-19 X-ray dataset from the Kaggle website, with 7395 images that consist of 3 classes (COVID-19, normal and pneumonia)	CNN-model with TL	An Xception Pre-Trained technique was used for feature extraction from the pre-processed image.	accuracy of 99.3%; sensitivity 99%; and specificity of 99% and F1-Score of 99.3%	There is no clinical investigation to support the suggested procedure’s dependability and efficacy. Therefore, it cannot yet replace a medical diagnosis provided by a licensed medical expert. As a result, a deeper investigation and a model developed using a substantially larger dataset are required.
[33]	The study aimed to detect COVID-19 using chest X-ray (CXR) images.	The radiographic images that was readily available was used in the study. The authors used a dataset of 10,040 samples, of which 2143 had COVID-19, 3674 had pneumonia, and 4223 were normal.	ResNet18 pretrained model was used	NA	The authors reported detection accuracy of 96.43%; sensitivity of 93.68%; area under the ROC curve was 99% for COVID-19. 97.0% for pneumonia, and 98.0% for normal cases	An improved outcome could be achieved with the introduction of feature selection or optimization technique
[76]	The research proposes a DL-based technique to predict the genome sequences of the SARS-Cov2 virus	SARSCov2 dataset	LSTM-RNN and GRU-RNN	Adam optimizer was used	LSTM-RNN achieved an accuracy and F1-score of 0.985 and 0.964 respectively, and GRU-RNN achieved an accuracy and F1-score of 0.987 and 0.945 respectively	The authors used optimizer in the study, but RNN technique might be responsible for low performance,
[74]	CBR model for cutting-edge reasoning tasks in the categorization of suspected COVID-19 instances	Italian SIRM repository dataset was used	Case-based reasoning (CBR) model	NA	94.54% accuracy was achieved	The used optimization technique might improved the performance of the model,
[21]	Proposed a rapid and valid method for COVID-19 detection using an AI technique	1020 CT slices from 108 patients with laboratory-proven COVID-19 and 86 patients with other atypical and viral pneumonia diseases were used.	ResNet-101	NA	Accuracy, 99.51%, sensitivity, 100%, specificity 99.02%	Kolmogorov-Smirnov test to determine whether all quantitative data are normal; two-tailed independent sample t-test and chi-square test to assess the age and gender distributions between COVID-19 and non-COVID-19 groups, respectively. There is no clinical investigation to support the study.
[46]	The study aimed at investigating the enforcement social distancing.	Covid-19 dataset	DL-techniques	NA	The detection accuracy of an infected person is up to 97%.	This technique was not used for diagnosis purposes, though the technique could be used for the detection of patients and contagious places.
[1]	Deep breathing sounds were proposed in the COVID-19 detection using the sound spectrum, image augmentation, and DL approaches.	COSWARA dataset and the Mel COCOA-2 augmented training datasets	DL-model called DeepShufNet was used	Mel spectrogram and GFCC.	A 90.1% accuracy, a 77.1% precision, a 62.7% recall, a 95.98% specificity, and a 69.1% f-score, respectively.	The proposed model showed the need for an improved model which may state-of-the-art methods for pandemic detection and prediction. However, the outcome shows the need for further investigation
[41]	The dataset was gathered with the help of an incredibly quick COVID-19 diagnostic sensor, and DL methods have been used.	Covid-19 dataset	CNN	NA	The findings suggest that SARS-CoV-2 samples may be correctly identified by the CNN algorithm with a 96.15% sensitivity 98.17% specificity and 97.20% accuracy respectively	By combining this DL-based model with the already-existing UFC-19, SARS-CoV-2 presence could be better detected. The outcome of the investigation shows that there is room for improvement which may be offer the introduction of optimization techniques
[16]	The paper compared the performance of sophisticated CNNs trained on chest CT images in detecting COVID-19 cases.	Patients with SARS-CoV-2 infections underwent 1252 scans, whereas patients with other lung disorders underwent 1230 scans. Additionally, 397 CT images without COVID-19 and 349 CT images of patients with COVID-19	ResNet101, Grad-CAM and DenseNet201	There was a visualization of the learning features extraction using the t-SNE technique	Accuracy 99.4%, 92.9%; Sensitivity 99.1% 93.7% and Specificity 99.6% 92.2% respectively	The identical image was copied to the three RGB channels, the size is changed without resizing, and feature vectors are visualized using distributed stochastic neighbor embedding.
[24]	The research aimed at detecting COVID-19 through DL technique using lung CT-SCAN images	SARS-COV-2 CT-Scan and Covid-CT Scan	MobileNet	NA	Accuracy 94.1% and Sensitivity 96.1%	Improvement of image resolution by residual dense block
[40]	The paper proposed optimization techniques for feature selection and classification of COVID-19	The authors used two datasets. The first is the COVID-19-dataset, which consists of 334 CT scans with COVID-19 clinical results. The second dataset, called non-COVID-19, contains an additional 794 CT scans of clinical patients without COVID-19.	AlexNet	Model optimization using Particle Swarm Optimization (PSO) feature selection using whale optimization algorithm (WOA)	Accuracy 79.0%, Sensitivity 81.0%, and Specificity 77.3%	Voting on several classifiers’ output using PSO and guided feature selection using WOA. The outcome of this experiment is poor and the prediction of future occurrence was not part of the study
[30]	Using affordable X-ray images to diagnose COVID-19 patients	X-ray images	multi-stream convolutional neural network model.	NA	With an accuracy of 97.76%, the findings are superior than those of other algorithms.	The outcomes also demonstrated that the suggested approach can significantly reduce the workload in health systems that is rapidly growing by using an AI-based automatic diagnosis tool. However, there is need for further research to improve the results
[84]	the coronavirus infection detection in lung imaging was investigated using current DL approaches.	5863 pediatric images from a lung image collection with three groups. Other dataset were RSNA Pneumonia Detection Challenge dataset and SIRM datasets Covid Chest X-ray dataset and Chexpert dataset	DWS-CNN + DSVM	Feature selection was	Accuracy 99.06%	The study achieved good results but the application of optimization algorithms can improve the performance of the system. The study was also limited to classification and no prediction
[34]	The paper proposed a unique deep CNN model that was inspired by Inception, replacing the Inception modules with depth-wise separable convolutions.	a larger images classification dataset with 17,000 classes and 350 million images	InceptionV3 ResNet50 ResNet101 ResNet152 InceptionResNetV2	NA	95.4, 96.1, 96.1, 93.4, 94.9%; 73.4, 76.5, 84.2, 74.8, 67.7%; 90.6, 91.8, 78.3, 65.4, 83.5% 96.0, 90.6, 98.2, 97.3, 95.4% 81.1, 83.5, 81.2, 69.8, 74.8% for accuracy, precision, sensitivity, specificity and F1-score respectively	The study achieved good results but the application of optimization algorithms can improve the performance of the system.
[25]	This paper presented a detection technique for COVID-19	Computed tomography (CT) chest images in the COVID-19 Radiography Database	CNN techniques	Bayesian optimization	For this structure, the metrics for sensitivity, precision, specificity, MCC, and F1-Score were 0.9642, 0.9642, 0.9812, 0.9641, and 0.9453, respectively.	The results of this study can be further improved with the introduction of better optimization and feature selection techniques
[36]	The study developed an hybrid model for detection the Progression of COVID-19 in patient	Clinical text data and chest X-rays dataset	VGG-16 (DL-based) model and logistic regression (ML-based) model were used.	NA	The model achieved an accuracy of 95%, precision of 97.2%, and *F*1 score of 97%.	The study used an hybrid method comprising DL and ML models, yet the detection and classification performance of the study was low, this might be liken to computational complexity on the dataset.
[69]	In this study, three pretrained cutting-edge CNN models were used to compare the detection of malware on IoT devices.	CT chest images in the COVID-19 Radiography Database	Inception-v3 CNN-based	NA	accuracy of 98.5% and 91%, respectively	The results of this study can be further improved with the introduction of better optimization and feature selection techniques
[48]	The study developed and test a new computer-aided diagnosis (CAD) for detection of coronavirus	chest X-ray images	CNN-based CAD scheme	NA	Accuracy 94.5%; Sensitivity 96.3% Specificity 97.2%	The study demonstrated that additional two image preprocessing steps and generating a pseudo color image in a DL CAD scheme. The outcome shows the need for further research to improve the performance.
[29]	The study proposed a few DL approaches to mitigate shipment risks by predicting “if a shipment can be exported from one source to another”, despite the restrictions imposed by the COVID-19	Online dataset from Kaggle	SoftMax, Random trees, RF, KNN, ANN, LSTM, RNN, TCN, BiLSTM	Feature extraction stage depends on two main variants of DL models	One of the TCN produce about 100% accuracy in predicting pandemic	There is no clinical investigation to support the suggested procedure’s dependability and efficacy. Therefore, it cannot yet replace a medical diagnosis provided by a licensed medical expert. As a result, a deeper investigation and a model developed using a substantially larger dataset are required.
[54]	The study presents the detection process of some diseases that are transmitted between animals and human beings to fight the diseases and reduce their spread	ImageNet data	Computer vision and Machine Learning with LSTM	NA	Precision, F1-measure	There was an improved outcome when compared with already existing works. However, utilization of bio-inspired optimization algorithms improved the results for large datasets.
[66]	In the task, authors compared DL and an ensemble techniques in the detection of COVID-19 in X-Ray images.	BIMCV-COVID19 dataset containing X-ray images, CT images and a bit of clinical data	DL- ensemble model	Adams optimizer	CLAHE + Single-image + end-to-end 87.59%, 69.31% and 72.34 for Training, validation and testing respectively. BCET + Multi-image + end-to-end 82.95%, 72.31% and 73.35 Training, validation and testing respectively.	Even after attempting numerous networks, regularization methods, and normalization techniques, the results are still subpar, never exceeding 80% accuracy in test. In this case, selection of relevant feature is suggested to improve the performance of the model.
[63]	The authors developed a unique end-to-end CNN model in conjunction with the residual network thinking and dilated convolution for the classification of COVID-19.	About 20,000 COVID-19 chest x-rays and CT scans images were used	VGG-19, ResNet-50 and InceptionV3 were used	NA	Vgg19, ResNet-50, and inceptionV3 attained an accuracies of 95.61%, 96.15%, and 95.16% respectively.	The study did not report other state-of-the-art metrics that might trade-off during the study. The accuracy of the prediction might also be improved when optimization techniques or feature selection algorithm is introduced
[26]	CoviWavNet was a DL-based pipeline for COVID-19 automatic diagnosis.	3D multiview dataset	ResNet CNN models	Deep spectral–temporal feature	Average accuracy of the three classifiers reached a final accuracy of 99.33%	The classification has been improved by the merging of deep spectral-temporal data with deep spatial features. The outcome gave room for better research in pandemic screening.
[39]	DL-based algorithms for classifying patients with COVID illness, healthy controls, and pneumonia classes.	Chest X-ray images	Temporal convolutional neural network (TCN)	SGDM and Adam’s optimizers	The results showed that RESCOVIDTCNNet exhibited the highest accuracy of 99.5% in classifying the three classes	One of the limitations of this study was that the number of images used in each class was too small and other state-of-the-art metrics were used to vali.
[37]	Proposed ensemble learning techniques for detecting pneumoconiosis disease in CXRs using multiple deep learning models	Chest X-ray radiographs (CXRs)	Deep ensemble learning	NA	The ensemble framework outperformed others, achieving an accuracy of 91.50% in the automated detection of pneumoconiosis.	Although, the ensemble learning techniques shown an improved performance but there is need for potential techniques to help radiologists in the primary screening of pandemic
[8]	A deep sequence learning-based technique to forecast improvement or worsening in subsequent chest X-ray scans using those scans was proposed	A lung disease and OVID chest X-ray datasets were used	DCNNs	NA	Among DCNNs feature extractors used, ChexNet (DenseNet121) PT outperformed others with Precision, recall, F-measure and AUC of 0.921, 0.918, 0.920, and 0.92 respectively	The accuracy of the prediction was not reported and other state-of-the-art metrics reported might be improved when optimization techniques or feature selection algorithm is introduced
[44]	The goal was to develop an uncertainty-aware CNN model for prediction of COVID-19	COVID19CXr, X-ray image, and Kaggle datasets	UA-ConvNet	EfficientNet-B3 model and Monte Carlo (MC) dropout	The suggested UA-ConvNet model achieves sensitivity of 98.15% and G-mean of 98.02% (with a Confidence Interval of 97.99–98.07).	The method demonstrated its superiority over the currently used techniques for identifying COVID-19 instances from CXR images. The outcome of the study might be improved when optimization techniques or feature selection algorithm is introduced
[31]	Chest X-ray image COVID-19 detection: A comparison of CNN architectures and ensembles	Covid-19 chest x-ray images	CNN and ensembles	NA	DenseNet169 produced the CNN instance results, with an accuracy of 98.15% and an F1 score of 98.12%. Further increases brought these numbers to 99.25% and 99.24%, respectively.	In order to recognize COVID-19 in CXR images, various CNN architectures were put to the test. The accuracy of the prediction might also be improved when optimization techniques or feature selection algorithm is introduced
[20]	The study proposed a DL-based technique called deep transfer learning, which can predict patients with Coronavirus disease automatically	Image of chest from: X-ray, Github and Kaggle	DL model	NA	In comparison to the other three models, the pre-trained ResNet50 model produced findings that were 98% accurate.	The study did not report other state-of-the-art metrics that might trade-off during the study. The accuracy of the prediction might also be improved when optimization techniques or feature selection algorithm is introduced
[83]	Authors aimed to identify the major SARS-CoV-2 population clusters	29,017 protein sequences of SARS-CoV-2 gene dataset from the NCBI viral database	convolutional autoencoder (CAE) that has been trained with numerical feature vectors mapped from coronavirus Spike peptide sequences.	NA	The clustering results revealed that there are six large SARS-CoV-2 population clusters (C1, C2, C3, C4, C5, C6). And C5 is the prevalence	The study focused on clustering using DL architecture with SARS-CoV-2 dataset.
[19]	The study suggests a brand-new method for detecting Dengue fever just from social media posts.	359,410 tweets dataset on infection between September 2017 and November 2019 were used.	RNN	n-gram and TF-IDF	Training accuracy 97.65%; accuracy 92.88%, precision 91.75%; 91.88%; F1-Score 91.88%	The study is NLP study, the authors analysed peoples’ comments which subject to bias and subjective. The model developed was not optimized and this might responsible to low performance of the detection model
[88]	A portable, precise, affordable, and user-friendly instrument that was driven by DL Isothermal Reverse-Transcription Loop-Mediated	COVID-19 dataset	KU-LAMP device with Deep learning	NA	the RT-LAMP output sample serves as a good modality to effectively screen COVID-19 via DL	There is no clinical investigation to support the suggested procedure’s dependability and efficacy.
[45]	An extensive and exhaustive guide to detect COVID-19 patient using Chest X-Ray samples	More than 460 COVID-19 chest images and 1266 Normal-CXR images dataset were used.	VGG InceptionV3 ResNet50 Xception	NA	InceptionV3 outperformed others with Accuracy, sensitivity and specificity of 95%, 90% and 97% respectively	The outcome of the study shows the need for further investigations as other tools or techniques might improved the outcome
[14]	An effective hybrid classification and segmentation method for COVID-19 detection based on CNN	Covid-19 CT images	hybrid CNN	NA	The study achieves classification accuracies of 100%, 99.45%, and 98.55%, respectively of different scenarios of the data for testing.	The obtained results demonstrated the effectiveness of the suggested method in aiding experts in automated medical diagnosis services. However, the outcome of the study might be improved if feature selection techniques or optimization techniques were used
[64]	The drone with DL capabilities was created for mask detection and social distance tracking.	After running the database create.py function, a dataset of image was collected for mask detection.	R-CNN model	NA	With Mask Precision, Recall and F1-Score 0.99; 0.86; 0.92; Without Mask Precision, Recall and F1-Score 0.88; 0.99; 0.93;	The detection accuracy for dataset with mask and without mask were not study. Also, the outcome of the study might be improved if feature selection techniques or optimization techniques were used.
[62]	A reliable and effective technique for COVID-19 early detection was suggested	A publicly open CXR image dataset were used	Deconvolutional Single Shot Detector (DSSD) which advancement of SSD and ResNet101	NA	an accuracy of 0.9597 and specificity of 0.9474 were achieved	The authors opined that model would help better decision-making for various aspects of detection and treat the infection. However, these results can be improved

DL-based techniques in contributing in no small measure to the pandemic control and healthcare delivery as a whole, it facilitates the detection, prediction, diagnosis and medical treatments of the infectious persons. Also, improvement in the applications for screening, recognition, segmentation, and classification across numerous areas of healthcare have been made possible by the use of DL techniques [68]. The results from the state-of-the-arts review indicates that, the use of optimization and feature selection approach was substantially efficient in terms of various performance in comparison to existing models DL techniques, but yet to be adopted in the detection and prediction of pandemic.

#### Limitations and challenges of DL techniques in the detection and prediction of pandemics

##### RQ4 What are the limitations of the existing DL techniques in the detection and prediction of pandemics?

The success of DL technology did not come without some challenges such as low detection and classification accuracy, which is a problem with pandemic detection and prediction [49]. Incomplete, improper label, unbalanced and biased dataset [78]. In addition, global challenges for the development of ML/DL/AI for clinical decisions are to be considered. Table 3 describes the main challenges for the detection and prediction of pandemics using the existing DL technology that have been noted in the literature. It is significant to note that each study in the literature has demonstrated the potential for automated pandemic detection and prediction but has also encountered difficulties or lacked thorough investigation and evaluation of the suggested solutions from many angles. It is worthy of note that COVID-19 pandemic dataset was the most prominent pandemic dataset that DL techniques were used to implement within the year under review, hence, the current survey is limited to COVID-19 pandemic studies.

**Table 3:** Limitation of DL techniques in the detection and prediction of pandemics

Ref	Limitations	Descriptions	Possible solution
[78]	Absence of quality dataset	Inaccurate, incomplete, improper label, unbalanced, biased dataset	Creation of annotated dataset for pandemic as well as supplying metadata are research directions and possible solution in this regard
[49]	The difficulty of interpreting the results to explain predictions	This flaw also creates a barrier to improving detection and classification accuracy, which is a problem with pandemic detection and prediction.	Creation of annotated dataset for pandemic as well as supplying metadata are research directions and possible solution in this regard
[84]	Absence of high-quality annotated data.	The performance of DL techniques cannot be enhanced without properly labelled, balanced, biased datasets and annotated high-quality data.	Creation of annotated dataset for pandemic as well as supplying metadata are research directions and possible solution in this regard
[38]	Failure of CNN model on certain dataset	There was a failure of CNN model on certain dataset which was undesirable, considering the critical nature of the pandemic reason for such a failure might be certainly results to the inefficiency and ineffectiveness of individual CNN models.	The designing or selecting better individual CNN architecture might help achieve better results. The introduction of feature selection or bio-inspired algorithms might improve the performance of the model.
[62]	Low model performance	Incomplete dataset, improper label, unbalanced This might be as a result of computational complexity	An attempt to create an ensemble-based strategy or hybridize some particular DL candidate models according to how well they have performed in the previous task under consideration, might improved the performance of the model. Modification of fully connected layers of DL model might improve the accuracy
[64]	Low accuracy	Limited dataset or non-selection of most relevant features might responsible for poor performance	To increase accuracy, future work can be expanded to address additional mask-wearing problems. Feature selection may also address the issue.

Different factors need to be considered to successfully implement the detection and prediction of a pandemic using DL techniques. In order to inform the research community and help it create more effective, reliable, and accurate DL models for the various challenges in the detection and prediction of pandemics, a critical analysis of the pertinent studies and techniques is conducted. The associated limitations are highlighted, and the research gaps and future challenges are identified. In order to inform the research community and help it create more effective, reliable, and accurate DL technique for the various limitations in the detection and prediction of pandemics, a critical analysis of the pertinent studies and techniques is conducted. The associated limitations are highlighted, and the research gaps and future challenges are identified.

### Outstanding issues in using deep learning solutions in pandemic diseases

DL-based techniques and architecture nevertheless have certain practical restrictions or downsides or gaps in their usage to predict and detect future pandemics. This paper highlights the limitations of DL-based techniques in the detection and prediction with respect to pandemics as scope for future research directions and the outstanding issues. These gaps include poor data quality management, difficulties in interpreting detection and prediction outcomes and low accuracy to mention but a few. One of the key findings of this survey is that most of the state-of-the-art studies did not apply optimization techniques, and/or feature selection in their studies, and few studies that applied either optimization or feature selection techniques did not predict of future occurrence of pandemics in the study. These issues may be addressed by developing hyper-parameter-based using bioinspired optimizer of DL techniques that modify the networks structures and the training process, ultimately develops DL-based architecture to give reliable first-hand detection and prediction of pandemics. The DL architecture’s parameters can be optimized using pre-trained model and this can also be compared with non-trained DL architectures. Additionally, by highlighting the crucial features, dataset improvement approaches can help increase detection accuracy. Overall, researchers, engineers, computer scientists and healthcare experts who working in the field of AI/DL/ML will find the results of this paper useful for development of an effective techniques for detection and prediction of pandemics to further prepare for future pandemic and IDs thereby helping healthcare delivery systems.

## Conclusion and future consideration

The previous and recent COVID-19 pandemic has inspired scientists to use various learning algorithm techniques to detect and predict the future occurrence of the pandemic. The current situation calls for a more accurate, more efficient, less complicated and inexpensive system that is capable of both the detection and prediction of pandemics and IDs. Although there are numerous diagnostic techniques for identifying pandemics such as SARS-CoV-2 infection, DL techniques happen to be one of the most widely used AI technologies to battle this pandemic. It has contributed in no small measure in curbing the pandemics as well as prevent its spread. This paper intends to find out various DL-based techniques and their contributions towards the detection and prediction of pandemics, Furthermore, this paper presents the state-of-the-art review of research activities about how AI/DL/ML techniques have contributed to the detection and prediction of pandemic in terms of pandemics monitoring systems, classification human activity recognition, data fusion, collecting vital signs of patients among others. In this survey, we reviewed more forty-four (44) published research papers written in a decade from 2013 to 2022 were reviewed. In order to understand the significance of DL contributions and limitations for the pandemic control. Hence, the current studies has contributed to the body knowledge by providing a comprehensive overview of the current state-of-the-art research in the field of AI particularly DL-techniques techniques for the detection and prediction of COVID-19 pandemic, identify gaps in the existing literature, and provide guidance for future research directions. This survey addresses and identifies a vacuum in the field by summarizing and assessing existing research that applied DL techniques for the detection and predictions of pandemics in their publication and identifies gaps in the existing literature for the purpose of future research direction in this aspect. An analysis of the different studies based on DL techniques for the detection and prediction of pandemic has been performed. The primary goal of this study is to provide researchers with some crucial research briefings that may help them create more effective and robust DL-based approach, that will be efficient and effective for the detection and prediction of pandemic. In order to explore the advantages of DL techniques better in the area of detection and prediction of pandemics and IDs control and prevention, this study considered varying challenges and alleviates them in different aspects such as feature selection, recognition, optimization and computational complexity.

## Data Availability

Data availability is not applicable to this study.

## Abbreviations

AI: Artificial intelligence
DL: Deep learning
DNN: Deep neural network
ML: Machine learning
CNN: Convolutional neural networks
COVID-19: Coronavirus
IDs: Infectious diseases
RT-PCR: *Reverse transcription polymerase chain reaction*
SARS: Severe acute respiratory syndrome
SARS-CoV-2: Severe acute respiratory syndrome coronavirus 2
SLR: Systematic literature review
WHO: World Health Organizations
